# Influence of CaCO_3_ on Density and Compressive Strength of Calcium Aluminate Cement-Based Cementitious Materials in Binder Jetting

**DOI:** 10.3390/ma17143463

**Published:** 2024-07-12

**Authors:** Tae-Hyung Kim, Bora Ye, Bora Jeong, Myeung-Jin Lee, Aran Song, Inkyung Cho, Heesoo Lee, Hong-Dae Kim

**Affiliations:** 1 Ulsan Division, Korea Institute of Industrial Technology (KITECH), Ulsan 44413, Republic of Korea; ttthkim@kitech.re.kr (T.-H.K.); yebora@kitech.re.kr (B.Y.); bora1106@kitech.re.kr (B.J.); leemj@kitech.re.kr (M.-J.L.); 2 Department of Material Science and Engineering, Pusan National University, Busan 46241, Republic of Korea; 3 Research Institute of Sustainable Development Technology, Korea Institute of Industrial Technology (KITECH), Cheonan 31056, Republic of Korea; arnsong@kitech.re.kr (A.S.); birdman98@kitech.re.kr (I.C.)

**Keywords:** CaCO_3_, calcium aluminate cement (CAC), binder jetting, powder bed density, compressive strength

## Abstract

We investigated the impact of CaCO_3_ addition on the density and compressive strength of calcium aluminate cement (CAC)-based cementitious materials in binder jetting additive manufacturing (BJAM). To confirm the formation of a uniform powder bed, we examined the powder flowability and powder bed density for CaCO_3_ contents ranging from 0 to 20 wt.%. Specifically, powders with avalanche angles between 40.1–45.6° formed a uniform powder bed density with a standard deviation within 1%. Thus, a 3D printing specimen (green body) fabricated via BJAM exhibited dimensional accuracy of less than 1% across the entire plane. Additionally, we measured the hydration characteristics of CAC and the changes in compressive strength over 30 days with the addition of CaCO_3_. The results indicate that the addition of CaCO_3_ to CAC-based cementitious materials forms multimodal powders that enhance the density of both the powder bed and the green body. Furthermore, CaCO_3_ promotes the formation of highly crystalline monocarbonate (C_4_AcH_11_) and stable hydrate (C_3_AH_6_), effectively inhibiting the conversion of CAC and showing compressive strengths of up to 5.2 MPa. These findings suggest a strong potential for expanding the use of BJAM across various applications, including complex casting molds, cores, catalyst supports, and functional architectural interiors.

## 1. Introduction

Binder jetting stands as an additive manufacturing process (BJAM), wherein organic or inorganic liquid binders are deposited onto a powder bed in a layer-by-layer. The efficacy of this process hinges significantly upon the powder bed density, a factor intricately linked to the characteristics of the 3D printing specimen (green body) [1,2]. Powder flowability, particle size distribution, and shape profoundly influence the packing density of the powder bed, thus directly impacting the green body’s properties [3].

Powder flowability, a key parameter, varies depending on external factors such as particle size [4], shape [5,6], and material composition [7]. Typically, spherical particles are preferred to ensure optimal flowability, with larger particles exhibiting superior recoating characteristics. Conversely, smaller particles, while enhancing surface quality and strength, may hinder recoating due to stronger intermolecular forces [8]. The incorporation of irregularly shaped powders poses challenges to flowability, yet slight compression during printing can enhance packing density [9,10]. Nevertheless, powders with low flowability can hinder the formation of a uniform powder layer during recoating, disrupting the binder pattern and ultimately compromising the density and compression strength of the green body. Multimodal mixtures, including bimodal or trimodal distributions, have demonstrated the ability to achieve higher packing densities compared to individual powders [11,12,13]. Moreover, there exists an optimal mixture ratio that maximizes the packing density. As the ratio of particle sizes in the mixture is adjusted, the maximum packing density also varies accordingly. In the case of bimodal alumina powder, a combination of 70 μm and 2 μm particles, a ratio of 70:30 vol% led to a significantly higher packing density of 82.65%. Furthermore, the use of a trimodal distribution, incorporating particles of sizes 70 μm, 10 μm, and 2 μm, demonstrated further improvement, achieving a packing density of 85.7% [14]. Consequently, careful consideration of powder flowability, particle size distribution, and shape becomes imperative. This is because they directly impact fluctuations in the density of the powder bed, thereby influencing the mechanical properties of the green body.

Utilizing powder binders such as cement [15,16] and gypsum [17,18] in the powder bed facilitates material bonding at low temperatures without sintering, offering energy efficiency advantages. When calcium aluminate cement (CAC) is incorporated with powder as a binder, it gains the advantage of rapid strength development within a day through a hydration reaction. In addition, ceramic BJAM technology can improve the performance of casting molds and catalyst supports because it is easy to manufacture complex 3-dimensional shapes and secure porous properties. Therefore, it has been applied in a variety of fields including casting mold, core, and catalyst supports [19,20,21,22,23]. However, CAC faces a significant decrease in compressive strength properties due to the conversion of the hydration product. As a result, its usage for general structural purposes is limited and predominantly employed in specialized applications such as marine structures, refractory products, and casting molds and cores, where attributes like heat and chemical resistance are paramount [24,25,26,27,28].

The widely recognized Portland cement (PC) and calcium aluminate cement (CAC) share a common trait: both react with water to produce insoluble hydrates [29,30,31,32]. However, CAC encounters a significant problem where its compressive strength sharply decreases due to conversion. During the conversion of metastable phases CAH_10_ and C_2_AH_8_ to the stable phase C_3_AH_6_, pores develop due to density differences, leading to a reduction in compressive strength. Typically, at a high water–cement ratio (w/c ≥ 0.7), nearly all anhydrous phases undergo an initial reaction with water to produce metastable hydrates. Conversely, maintaining a low water–cement ratio (w/c ≤ 0.4) means there is not enough water for all anhydrous phases to react and form metastable hydrates. The water released during subsequent conversion hydrates the unreacted anhydrous phases, potentially resulting in a denser microstructure [33]. Furthermore, temperature significantly influences the conversion of CAC. Specimens of CAC cured at w/c = 0.5 for 1 to 300 days and kept at low temperatures (≥20 °C) only form low-density hydrates across all specimens. However, in specimens subjected to 60 °C for the initial hours of hydration, a stable microstructure comprising solely the stable phases AH_3_ (gibbsite) and C_3_AH_6_ (hydrogarnet) emerges [34].

Luz et al. (2012) investigated the application of CAC in endodontic treatments by incorporating CaCO_3_ of approximately 1 μm, examining the hydration reactions and changes in mechanical strength. The results indicated that the addition of 14 wt.% CaCO_3_ generated sufficient C_3_A·CaCO_3_·11H (C_4_AcH_11_) to suppress the formation and conversion of metastable hydrates (CAH_10_, C_2_AH_8_), thereby alleviating conversion and significantly enhancing mechanical strength and dimensional stability. Furthermore, the quantitative analysis of X-ray diffraction results provided insights into how the hydration phases affect mechanical properties, particularly compressive strength [29]. Despite various studies on CAC, research on the characteristics (particle size, shape, and powder flowability) of CAC-based cementitious powders for BJAM and their behavior in the powder bed is limited. Moreover, detailed investigations focusing on the compressive strength and density of CAC-based cementitious powders in BJAM, considering powder and powder bed characteristics, are scarce.

Therefore, this study aims to address these technical gaps by investigating the density and compressive strength of CAC-based cementitious powders in BJAM. We propose a high-precision method for measuring powder bed density and aim to understand the formation of powder bed density by examining powder characteristics such as particle size distribution, particle shape, and powder flowability. Additionally, the study will investigate how the addition of CaCO_3_ affects the density and compressive strength of the powder bed in CAC-based cementitious materials used in BJAM.

## 2. Materials and Methods

### 2.1. Preparation

In this study, spherical silica (s-SiO_2_, LUOYANG TONGRUN INFO TECHNOLOGY CO., LTD., Luoyang, China) was utilized to achieve uniform powder bed formation in BJAM. As powder binders, alumina cement (CaAl_2_O_4_, Union Corp., Seoul, Republic of Korea) and dodecacalcium hepta-aluminate (C_12_A_7_, Union Corp., Seoul, Republic of Korea) were employed. For smooth jetting of the activator, 2 wt.% polyethylene glycol (PEG, Purity: >99%, Daejung Chemicals & Metals Co., Ltd., Siheung, Republic of Korea) and 1 wt.% glycerol solution (Purity: >99%, Sigma Aldrich, St. Louis, MO, USA) were added to deionized water. Calcium carbonate (CaCO_3_, Purity: >99.0%, Sigma Aldrich, USA) was used as received from the manufacturers. The w/c ratio was determined by averaging the amount of liquid activator measured in Z print software (ver 7.6) and the amount of water calculated from the green body. The mixing ratios for each material are summarized in Table 1.

The powder materials for BJAM were prepared following these steps: initially, s-SiO_2_, CaAl_2_O_4_, C_12_A_7_, and CaCO_3_ powders were weighed according to the mixing ratio. Then, they were placed in a cylindrical plastic container and mixed by rotation at 60 rpm for 24 h. The mixed powder was sieved through a 140-mesh sieve to obtain the final powder. The control specimen did not contain CaCO_3_. Based on the CaCO_3_ content, specimens containing 10 wt.%, 15 wt.%, and 20 wt.% were named 10 Cc, 15 Cc, and 20 Cc, respectively. The preparation of the activator involved adding 2 wt.% polyethylene glycol and 1 wt.% glycerol solution to deionized water in a 500 mL beaker. The mixture was stirred at 25 °C for 1 h to obtain the final activator solution.

### 2.2. Binder Jetting Additive Manufacturing Process (BJAM)

**Figure 1:**
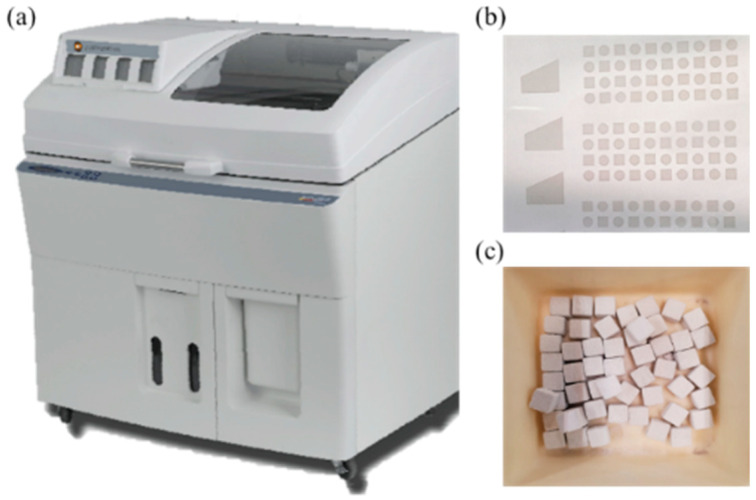
Image of (**a**) binder jet 3D printer, (**b**) powder bed surface deposited by activator, (**c**) green bodies fabricated via BJAM.

Figure 1 illustrates (a) binder jet 3D printer (Spectrum Z510, Z Corp., Rock Hill, SC, USA), (b) powder bed surface deposited by the activator, and (c) green bodies fabricated via BJAM. The specifications of the binder jet 3D printer utilized in the experiment are presented in Table 2. The 10 × 10 × 10 mm^3^ cubic specimens were fabricated to analyze the hydration behavior induced by the addition of CaCO_3_. All specimens produced via BJAM were air-dried for 1 day after printing, followed by de-powdering to obtain the green body.

### 2.3. Measurement for Powder Bed Density

**Figure 2:**
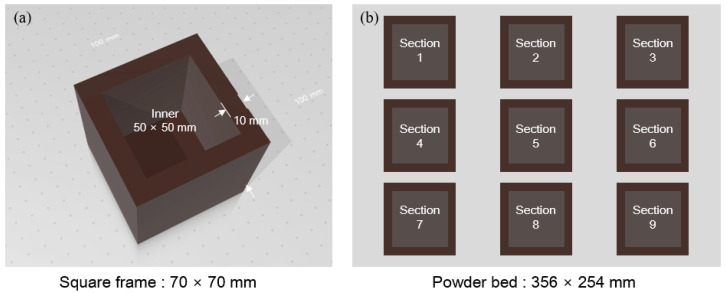
Methodology of powder bed density, (**a**) designed square frame, (**b**) arrangement of square frame in the powder bed.

Researchers have attempted to infer the powder bed density through powder characteristics such as bulk density, tap density, and Hausner ratio. However, it remains uncertain whether any of these powder characteristics can accurately predict the powder bed density [1]. In this study, the powder bed is recoated using the roller spread method. To measure the density of the powder bed, it was divided into 9 sections and a square frame with an outer wall thickness of 10 mm and an inner dimension of 50 × 50 × 50 mm^3^ was printed (Figure 2). No activator is deposited inside the square frame. The square frame produced to measure the density of the powder bed was dried in the powder bed at 25 °C for 1 day, then carefully recovered to prevent the inner powder from escaping, and the outer powder was removed. The internal powder of the square frame was recovered and weighed using an electronic balance (AR1140 Adventurer, resolution: 0.1 mg). Because the powder was recovered with a brush, there is a measurement error due to the separation of some powder particles. The density of the powder bed was calculated using the following Formula (1):

Powder bed density (ρ_pb_) = (M_pb_/V_pb_) (1)

where M_p_ is the recovered powder mass measured from inner square frame. V_pb_ is the inner volume of the square frame (50 × 50 × 50 mm^3^).

### 2.4. Measurement of Green Body Density

The density of the green body cannot be measured using the conventional Archimedes method for a 3D-printed specimen (green body). This is because the hardened binder would dissolve in the fluid, compromising the integrity of the green body. Additionally, in cement-based materials, there can be additional hydration of unreacted clinker. Therefore, green body density was measured using a geometric density approach [29]. In this approach, the mass of density measurement specimen (10 × 10 × 10 mm^3^), measured using an electronic balance (AR1140 Adventurer, resolution of 0.1 mg), was divided by the volume measured with a digital caliper with a resolution of 0.01 mm. To ensure accuracy, the measurements were repeated 10 times, excluding the minimum and maximum values, and averaged for calculation.

### 2.5. Characterization

For particle size distribution analysis of raw powders, PSA (Particle size distribution analysis, Mastersizer 3000, Malvern Panalytical, Great Malvern, UK) was conducted. XRD (X-ray Diffractometer, D8ADVANCE CuK = 1.5406 Å, Bruker, Billerica, MA, USA) analysis was performed to confirm crystalline phases of the raw materials and the hydration phases of CAC. Testing conditions involved a scan speed of 5°/min in the 2θ range of 10–55° with a measurement interval of 0.02°. Thermal decomposition behavior of CAC hydrate phases and carbonates was determined via thermal gravimetric analysis (TGA 2, METTLER TOLEDO, Columbus, OH, USA). The analysis was carried out in an air atmosphere within the temperature range of 25–800 °C at a heating rate of 10 °C/min, with a mass of 50 ± 5 mg placed in a 70 μL alumina crucible to ensure uniform analysis. Uniaxial compressive strength was measured using a digital universal testing machine (FGP-100, Nidec-Shimp Corp, Glendale, CA, USA). For each experimental group, five specimens for compressive strength were tested, and the average value was recorded after excluding the maximum and minimum values. Dimensional precision of the 3D-printed specimens was determined using an electronic vernier caliper, measuring in the X, Y, and Z axes, dividing the total length into thirds, conducting 3 measurements for each axis, and recording the average value. Dimensional accuracy was measured for five specimens per experimental group to obtain the data. To evaluate the powder flowability, a dynamic powder flow analyzer (Revolution, Mercury Scientific Inc., Newtown, CT, USA) was used. All specimens were weighed using a precise electronic scale (AR1140 Adventurer balance, Ohaus Corp., Parsippany, NJ, USA).

## 3. Results and Discussion

### 3.1. Powder Characterization

**Figure 3:**
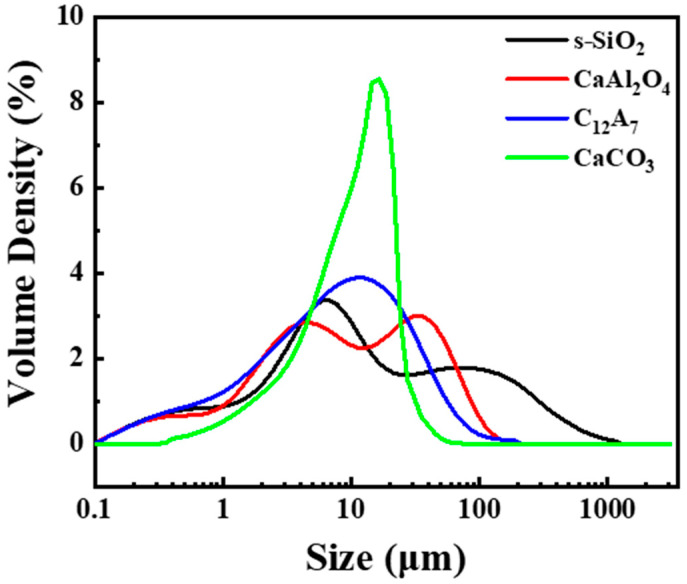
Particle size distribution of s-SiO_2_, CaAl_2_O_4_, C_12_A_7_, CaCO_3_ for BJAM.

Particle size distribution analysis for raw powders used in this experiment is presented in Figure 3 and Table 3. Typically, mixing powders with varying particle sizes enhances the density of the powder bed, leading to an improved density of the green body [11,12,13]. Spherical silica (s-SiO_2_) was measured with a d_50_ of 31.2 μm and possesses spherical morphology, enabling it to form a uniform powder bed in BJAM due to its flowability [8]. Alumina cement particles, acting as powder binders, were measured with a d_50_ of 10.7, 12.1 μm, indicating their ability to fill spaces between CAC-based cementitious particles and facilitate inter-particle bonding through hydration reactions. The role of CaCO_3_, with a d_50_ of 19.3 μm, is expected to fill spaces between particles, thereby enhancing powder bed density.

**Figure 4:**
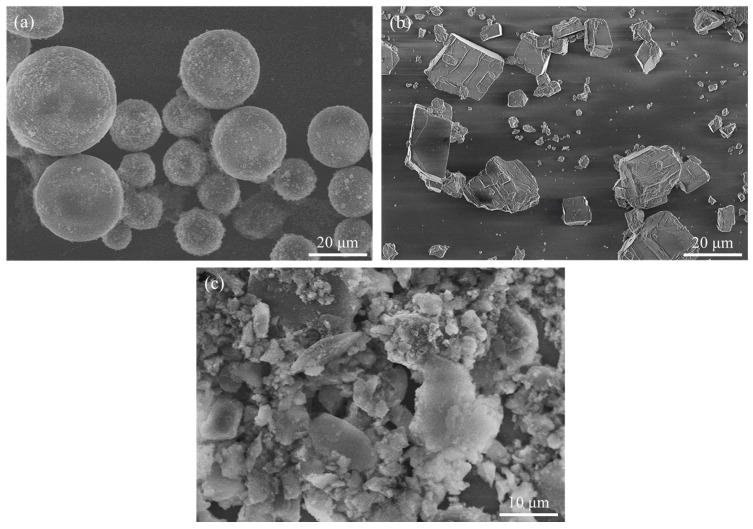
SEM images of raw powders for BJAM (**a**) s-SiO_2_, (**b**) CaCO_3_, (**c**) alumina cement (CaAl_2_O_4_) and dodecacalcium hepta-aluminate (C_12_A_7_) powder mixture.

Figure 4 shows the particle shapes of raw materials for BJAM. Silica is observed to be perfectly spherical shaped. Spherical particle shapes are suitable for powder bed-based additive manufacturing due to their high flowability, which results from low inter-particle friction. Calcium carbonate exhibits a cubic shape typical of calcite, while alumina cements CaAl_2_O_4_ and C_12_A_7_ show irregular particle shapes. These non-spherical particles in calcite and alumina cements can hinder powder flowability, making it essential to conduct flowability tests and verify the formation of a uniform powder bed.

**Figure 5:**
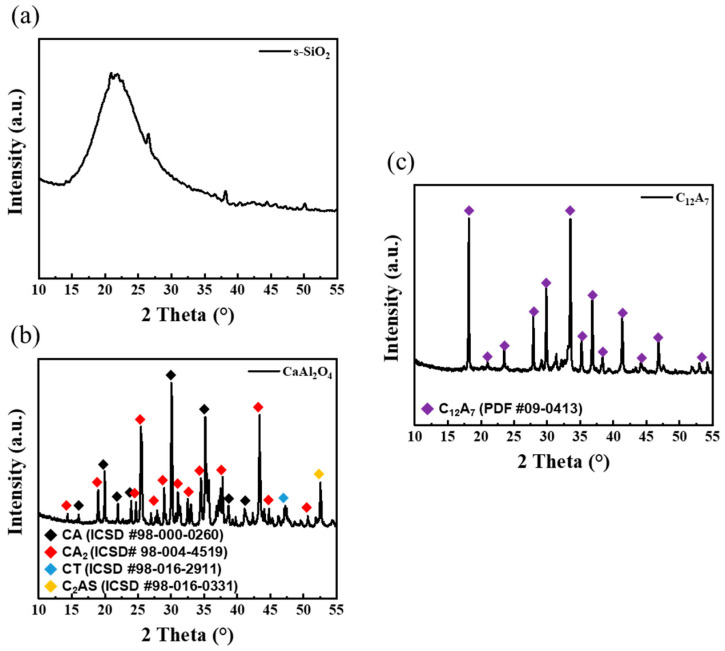
X-ray diffraction patterns of raw materials (**a**) s-SiO_2_, (**b**) alumina cement (CaAl_2_O_4_), (**c**) Dodecacalcium hepta-aluminate (C_12_A_7_).

Figure 5 illustrates X-ray diffraction patterns of s-SiO_2_ and CaAl_2_O_4_ and C_12_A_7_. The s-SiO_2_ exhibits typical broad peaks characteristic of amorphous silica (Figure 5a) [30]. And the X-ray diffraction pattern of CaAl_2_O_4_ in Figure 5b predominantly shows the presence of anhydrous clinker phases CA (ICSD #98-000-0260) and CA_2_ (ICSD #98-004-4519), with traces of CT (ICSD #98-016-2911) and C_2_AS (ICSD #98-016-0331) phases observed. In Figure 5c, C_12_A_7_ (PDF #09-0413) displays a characteristic phases of dodecacalcium hepta-aluminate [31].

**Figure 6:**
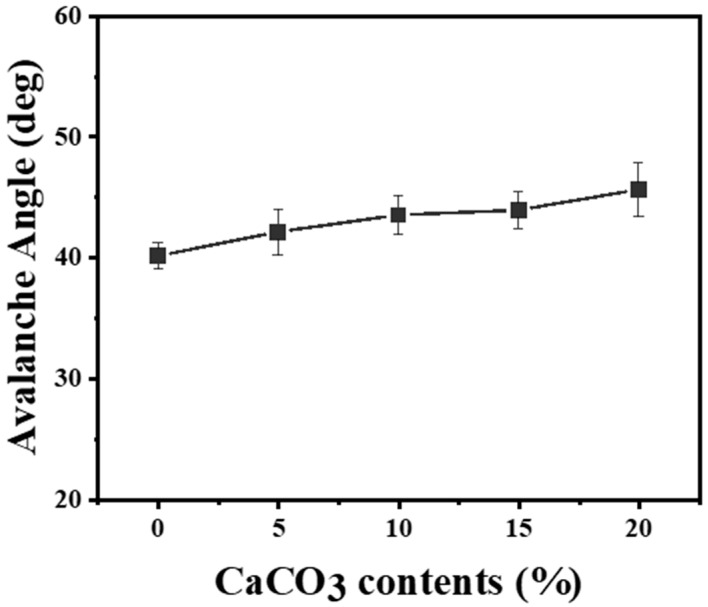
Powder flowability (avalanche angle) depends on CaCO_3_ contents ranging from 0–20 wt.%.

**Figure 7:**
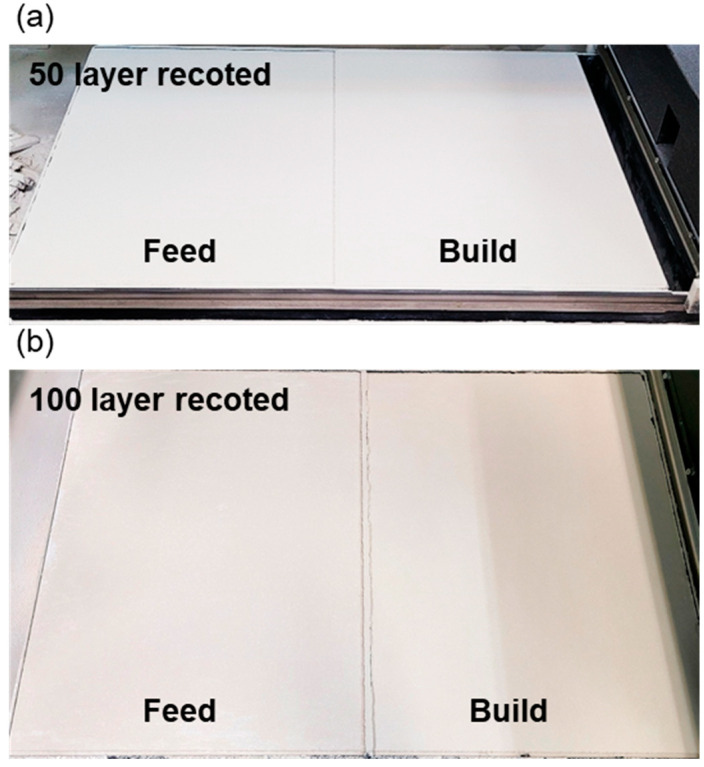
Powder bed surface formation using 20 Cc powder mixture (**a**) after 50 layer recoated (**b**) 100 layer recoated.

Measuring powder flowability as an avalanche angle is a more suitable methodology in powder bed-based additive manufacturing than other techniques [32]. We used a dedicated container to measure the tapped volume of 100 cm^3^. A caontainer was filled with powder until it could no longer hold any more, then tapped and leveled to remove excess powder. Subsequently, powder was transferred to a cylindrical drum with a transparent glass window. The drum was designed to rotate at 0.3 rpm to measure the pure powder flow energy. The conditions for measuring the avalanche angle involved averaging data over a total of 150 occurrences of avalanches, recorded at a rate of 30 fps. Figure 6 shows the change in avalanche angle with respect to the content of CaCO_3_. It increased as the CaCO_3_ content increased from an initial avalanche angle of 40.1°. The addition of 20 wt.% CaCO_3_ led to a significant increase in the avalanche angle to 45.6°. Figure 7 depicts the powder bed surface after recoating 50 and 100 layers using the 20 Cc powder with an avalanche angle of 45.6°. Even after repeated recoatings of 50 and 100 layers, no significant aggregation or large defects on the powder bed surface were observed. SY Chun et al. (2023) conducted a study on the recoating speed that affects the quality of the powder bed surface, depending on cement content used as a powder binder. When cement content was increased to 30 wt.%, it exhibited an avalanche angle of 45.2°, limiting the recoating speed that does not affect surface quality to 50 mm/s [33]. However, in our study, no defects were observed on the powder bed surface even with a powder mixture having an avalanche angle of 45.6° and a recoating speed of 100 mm/s. Therefore, further investigation is needed regarding the dimensional precision and density of the powder bed surface quality.

**Figure 8:**
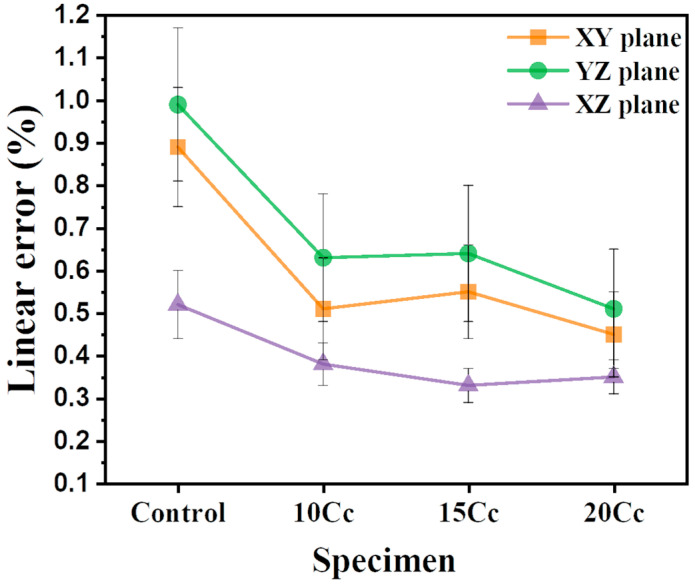
Dimensional accuracy results on XY, YZ, and XZ planes for green bodies fabricated via BJAM.

Figure 8 illustrates the dimensional precision of the XY, YZ, and XZ planes for green bodies. To verify dimensional accuracy changes due to the presence of CaCO_3_ incorporation, layer thickness and saturation level used in printing were fixed at 102 μm in Zprint software (ver 7.6). For the control green body specimen, where CaCO_3_ was not added, the linear errors were measured as 0.89 ± 0.14% for the XY plane, 0.99 ± 0.18% for the YZ plane, and 0.52 ± 0.08% for the XZ plane. The linear errors in the XY and YZ planes may relatively increase due to the spreading of the liquid activator into the powder bed. As the content of CaCO_3_ increased, the dimensional accuracy of the green bodies containing CaCO_3_ improved. The specimen of 20 Cc exhibited the highest dimensional accuracy among them, attributed to the increase in powder bed density because of mixing different materials with varying particle size distributions. Dimensional accuracy for the 20 Cc green body was improved to 0.45 ± 0.10% for the XY plane, 0.51 ± 0.14% for the YZ plane, and 0.35 ± 0.04% for the XZ plane. The dimensional accuracy of the 15 Cc and 20 Cc green bodies was nearly at the same level.

Notably, a dimensional accuracy of less than 1% across the entire surface is a significant achievement in ceramic binder jetting using multimodal powders. Using the bimodal powder of dextrin-coated Al_2_O_3_ with 69.6 vol% of d_50_ = 0.82 μm and 30.4 vol% of d_50_ = 18.5 μm, the fabricated green body showed dimensional errors of up to 6.7% in Z dimension compared to the designed 3D geometric model (50 × 8 × 6 mm^3^) at a layer thickness of 100 μm and a saturation level of 110% [34]. Therefore, results obtained while fixing the saturation level and layer thickness indicate that the dimensional accuracy of the green body may be improved by the multimodal powder effect.

### 3.2. Powder Bed and Green Body Density

Table 4 presents densities of the powder bed measured using a square frame. The inner height of the square frame used for measuring powder bed density is 490 layers. As the content of CaCO_3_ increased, the density of the powder bed increased, and the standard deviation decreased. There was not a significant difference in the powder bed density among the central section (section 5), edge sections (section 1, 3, 7, 9), and mid-edge sections (section 2, 4, 6, 8) in all experimental groups. Therefore, powder with an avalanche angle value between 40.1° and 45.6° ensures the formation of a uniform powder bed.

Green body density was measured using the following equation:

Green body density (ρ_gb_) = M_gb_/V_gb_ (2)

ρ_gb_ represents the measured green body density, M_gb_ is the weight of the powder recovered within the inner area of square frame, and V_gb_ is the volume within the inner square frame.

**Figure 9:**
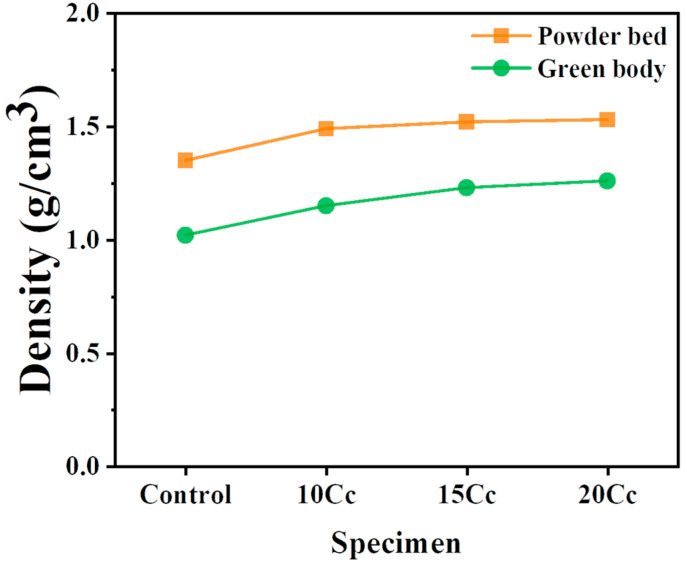
The correlation between the powder bed density and green body densities fabricated via BJAM.

Figure 9 illustrates the measured density of the powder bed and the green body. Results from all specimens indicate that the powder bed density is higher than the green body density. The green body density of the control specimen was measured at 1.02 g/cm^3^, while for specimen containing CaCO_3_, it improved to 1.15, 1.23, and 1.26 g/cm^3^, respectively. The increase in green body density is attributed to the packing effect of the powder layer. This is because irregularly shaped alumina cement particles (CaAl_2_O_4_, C_12_A_7_) and CaCO_3_ fill the spaces between the spherical silica particles. Moreover, a mixed powder system with irregular particle shapes can also benefit from the compaction effect exerted by the recoating roller. Additionally, the filling effect can be enhanced by the mixing of powders with different particle size distributions. Therefore, this aligns with the concept that a higher powder bed density can enhance the green body density.

Despite significant efforts, predicting the powder bed density of new powders remains challenging. Furthermore, directly measuring the powder bed density of new powders is not preferred due to increased costs and labor. An experimental study of the relationship between powder characteristics (apparent density, tap density, and Hausner ratio) and powder bed density was reported using seven different sizes of spherical alumina powders ranging from 0.05 μm to 70 μm. According to the research findings, apparent density was suggested as a strong indicator for predicting powder bed density [1]. Nevertheless, due to the various conditions (mixtures of multiple compositions, multimodal particle size distributions, and various particle shapes), there are limitations to accurately predicting powder bed density based solely on powder characteristics. Therefore, while the square frame method proposed in this experiment may not be the most efficient way to measure powder bed density, it underscores its high accuracy.

### 3.3. Crystalline Phase Analysis

**Figure 10:**
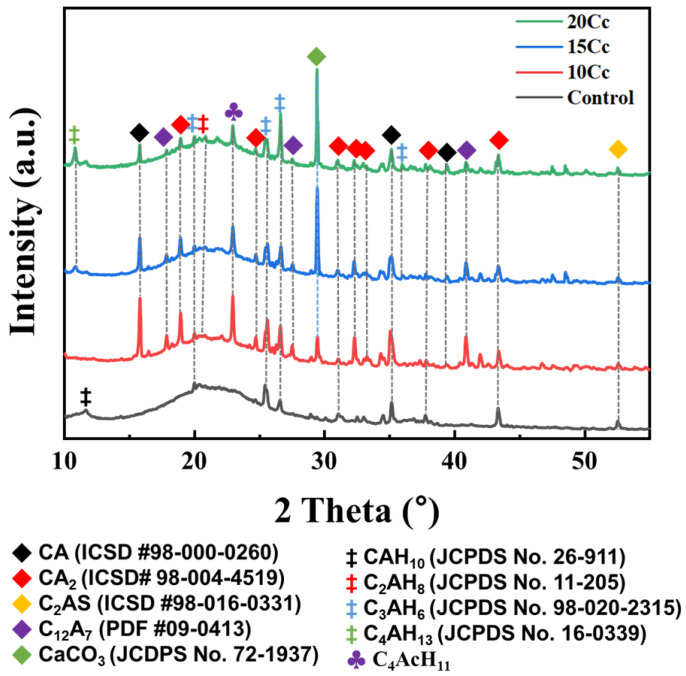
X-ray diffraction patterns of hydrated alumina cement phases for 3D-printed specimens with varying CaCO_3_ content, dried for 1 day at 25 °C, C_4_AcH_11_ [35,36].

Figure 10 depicts X-ray diffraction patterns of green bodies of the entire specimen. Typically, well-known hydration reactions of CAC generally occur according to Reactions (3)–(6). Reactions (3) and (4) involve hydration of anhydrous CAC clinker with water to form metastable hydrates, while Reactions (5) and (6) represent the conversion of these metastable phases into stable hydrated phases.

CA + 10H → CAH_10_ (3)

2CA + 16H → C_2_AH_8_ + AH_3_ (4)

2CAH_10_ → C_2_AH_8_ + AH_3_ + 9H (5)

3C_2_AH_8_ → 2C_3_AH_6_ + AH_3_ + 9H (6)

The control specimen illustrates an X-ray diffraction pattern without CaCO_3_, revealing the presence of a low density hydration phase (CAH_10_) at around 11.8°. Additionally, abundant unreacted anhydrous clinker phases and small amounts of C_2_AH_8_ and C_3_AH_6_ are observed. With the green body of 10 Cc, a diffraction peak of CAH_10_ disappears, and stable phase (C_3_AH_6_) sharply emerges. This was confirmed to occur because the C_4_AcH_11_ layered hydrate interferes with the conversion of CAC [35,36].

3CA + Cc + 17H → C_4_AcH_11_ + 2AH_3_ (7)

It is noted that production of C_4_AcH_11_ does not appear to increase proportionally with the amount of CaCO_3_. Furthermore, as the intensity of the diffraction peak of C_4_AcH_11_ increases, the intensity of diffraction peaks of unreacted CAC clinkers (CA, CA_2_, C_12_A_7_) also increases. Thus, it is confirmed that in the hydration reaction of CAC with CaCO_3_, an increase in unreacted CAC clinker phases occurs due to the greater generation of C_4_AcH_11_.

As the CaCO_3_ content increases to 10–20 wt.%, a distinctive diffraction pattern of CaCO_3_ (calcite) becomes evident at 29.4°. For CAH_10_, no presence is observed in specimens containing CaCO_3_, while for C_4_AH_13_, there is an increasing trend in specimens with 15 Cc or more CaCO_3_ contents. C_4_AH_13_ is known to increase porosity as it transforms into stable hydrogarnet at room temperature [37]. Therefore, based on the X-ray diffraction pattern results, 10 wt.% CaCO_3_ addition would be optimal contents for hindering the conversion of CAC, as it exhibits minimal presence of C_4_AH_13_ and the highest peak of C_4_AcH_11_. The diffraction pattern of C_2_AS (ICSD #98-016-0331) remains unchanged with the addition of CaCO_3_, indicating rare impact.

### 3.4. Compressive Strength Evolution

**Figure 11:**
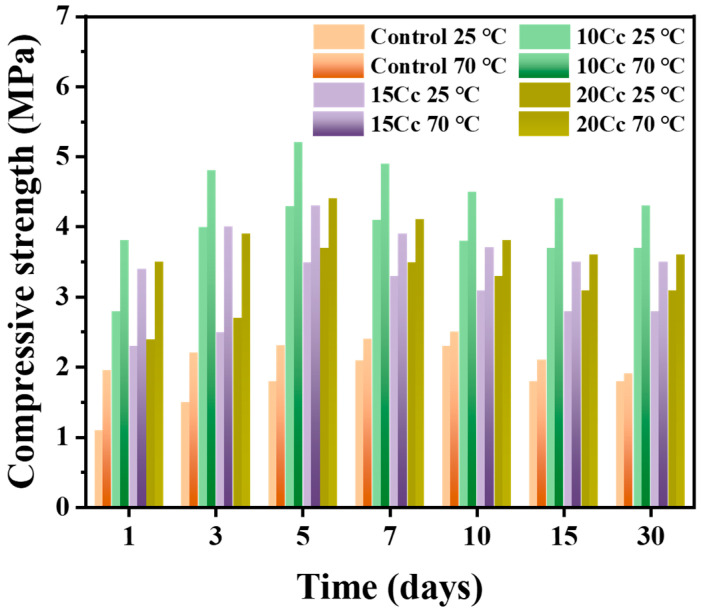
Measurement of changes in compressive strength over 30 days for 3D-printed specimens, dried at 25 °C and 70 °C.

Figure 11 illustrates variation in compressive strength with varying CaCO_3_ contents. Overall, the change in compressive strength follows a pattern of initially increasing to reach maximum strength and then decreasing while maintaining consistency. This trend aligns well with the conversion of CAC. The control specimen showed the highest compressive strength, reaching 2.3 MPa at 25 °C and 2.5 MPa at 70 °C after 10 days, with a subsequent decrease of approximately 21% and 24% after 30 days. In the green body of 10 Cc, a significant enhancement in compressive strength was observed, reaching 4.3 MPa at 25 °C and 5.2 MPa at 70 °C after 5 days, followed by a decrease of approximately 14% and 17% after 30 days.

Okpin Na et al. (2021) demonstrated results with a compressive strength of up to 7 MPa using binder jet 3D printing based on CSA cement materials [38]. In this study, the 10 Cc green body exhibited a maximum compressive strength of 5.2 MPa, indicating relatively lower mechanical properties. However, when comparing dimensional precision, we achieved excellent precision of less than 1%, whereas the reported CSA cement material-based binder jet 3D-printed specimens had precision in the range of 5–10%. Therefore, we judge that our results are superior, not only due to high precision but also because we achieved a compressive strength exceeding 5 MPa.

A notable point is that the addition of 10 wt.% CaCO_3_ resulted in a 30% improvement in the rate of decrease in compressive strength compared to the control specimen. These improvements could be attributed to the formation of mono-carbonate (C_4_AcH_11_) and stable hydration products (C_3_AH_6_), as indicated by X-ray diffraction patterns [35,36]. Furthermore, reduction in the time to achieve maximum strength from 10 days to 5 days suggests that the addition of CaCO_3_ can accelerate the hydration reaction. These results are consistent with the trend where the maximum compressive strength is achieved after 5 days of drying for all specimens containing CaCO_3_. Green bodies of 15 Cc and 20 Cc exhibited a similar rate of decrease in compressive strength. These results support the existence of an optimal CaCO_3_ content that can prevent the conversion of CAC, indicating the inhibitory effect of mono-carbonate (C_4_AcH_11_) on the conversion of CAC.

### 3.5. Thermogravimetric Analysis

**Figure 12:**
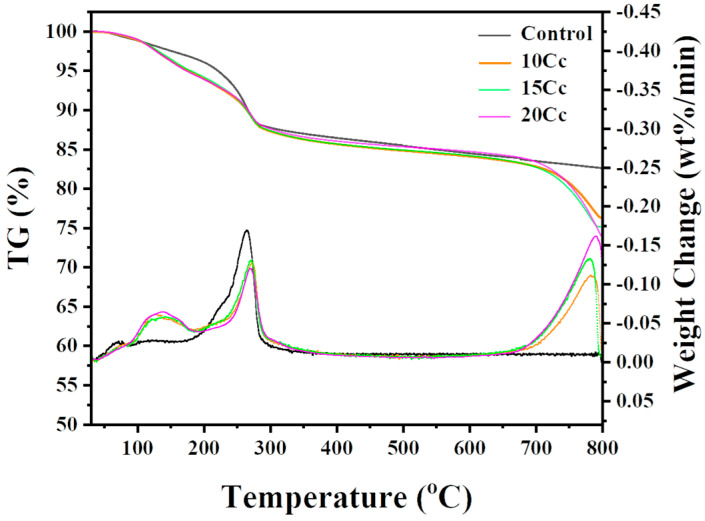
Thermal decomposition behavior (TG/DTG) of hydrated alumina cement phases for 3D− printed specimens, dried for 1 day at 25 °C.

Figure 12 illustrates the TG/DTG curves of entire specimens. Characteristic temperature ranges of thermal decomposition for CAC hydrates and carbonates are as follows [35,39]. It has been reported that CAH gel, CAH_10_, and C_2_AH_8_ exhibit thermal decomposition peaks in the range of 20–180 °C. Additionally, peaks due to the dehydration of free water can overlap at temperatures below 100 °C. In the control specimen, peaks were observed below 130 °C, which were attributed to the dehydration of CAH gel, CAH_10_, C_2_AH_8_, and free water. Specimens containing CaCO_3_ exhibit a single peak in the range of 100–180 °C. Further peaks associated with C_2_AH_8_ can occur in the range of 180–220 °C, but these were not observed in the control specimens. Stable hydrates undergo dehydration in the range of 220–370 °C, corresponding to AH_3_ and C_3_AH_6_. AH_3_ exhibits the highest peak near 260 °C, C_3_AH_6_ shows the highest peak in the range of 290–370 °C. Decomposition corresponding to carbonates (CaCO_3_) occurs at temperatures above 650 °C.

Notably, entire specimens containing CaCO_3_ exhibit a single peak in the range of 100–180 °C, corresponding to the formation of C_4_AcH_11_. However, weight loss of thermally decomposed C_4_AcH_11_ was nearly identical across the specimens. Based on these results, it can be inferred that the amount of C_4_AcH_11_ generated through the hydration reaction does not directly correlate with the amount of CaCO_3_. Nevertheless, XRD and compressive strength results indicated that the specimen containing 10 wt.% CaCO_3_ exhibited the highest crystallinity and compressive strength. From these findings, it can be concluded that increased crystallinity of C_4_AcH_11_ may enhance the inhibitory effect on the conversion of CAC.

## 4. Conclusions

Based on these findings, several conclusions can be drawn regarding the influence of CaCO_3_ on the density and compressive strength of CAC-based cementitious materials in BJAM:

**Powder Bed Density and Flowability**: The square frame method proposed in this experiment allows for highly accurate measurement of powder bed density. This method does not rely on predicting the characteristics of powder but rather represents the most realistic values. Furthermore, the standard deviation of powder bed density in the entire experimental group was approximately 1%, indicating the formation of a homogeneous powder bed. Also, the addition of CaCO_3_ increases powder bed density. This improvement is due to the filling of finer CaCO_3_ particles between the CAC-based cement particles, contributing to the densification of the powder bed.

Powder flowability, indicated by the avalanche angle, plays a crucial role in ensuring the formation of a uniform powder bed in powder bed-based additive manufacturing processes. Powders with avalanche angles ranging from 40.1–45.6° can form a uniform powder bed surface without significant agglomeration or defects. These powder bed density results, with a standard deviation of approximately 1%, support the formation of a homogeneous powder bed.

**Dimensional Accuracy of Green Body**: The dimensional precision of the green body improved proportionally with CaCO_3_ content. Compared to the control specimen, the overall average dimensional error for the 20 Cc green body improved from 0.80% to 0.44%. This improvement can be attributed to the increased powder bed density achieved through the addition of CaCO_3_, which promotes the formation of a uniform and compacted green body during BJAM. Notably, achieving an average dimensional error of less than 1% across the entire surface is a significant accomplishment for ceramic BJAM using multimodal powders.

**Crystallographic Analysis and Compressive Strength**: According to the analysis of X-ray diffraction patterns, the presence of CaCO_3_ induces the formation of C_4_AcH_11_ during hydration reactions, minimizing the formation of metastable phases (CAH_10_, C_2_AH_8_) and promoting the development of a stable and dense microstructure. The 10 Cc green body exhibited up to a 208% improvement in compressive strength compared to the control specimen and showed a 30% enhancement in mitigating the property reduction due to the conversion effect. This increase in compressive strength is attributed to CaCO_3_’s role in promoting the formation of monocarbonate (C_4_AcH_11_) and high-density hydrate (C_3_AH_6_).

The crystallinity of C_4_AcH_11_, which significantly influences compressive strength, varied with the CaCO_3_ content and was found to be highest in the 10 Cc green body. These results suggest that there is an optimal CaCO_3_ content for enhancing mechanical properties, identified in this study as 10 wt.%.

**Thermal Analysis (TG/DTG)**: The presence of CAC hydrates and carbonates was confirmed across all specimens. Notably, all specimens containing carbonates exhibited a single peak in the 100–180 °C range, corresponding to the formation of C_4_AcH_11_. However, the weight loss from the thermal decomposition of C_4_AcH_11_ within this temperature range was nearly identical across all specimens. Based on these results, it can be inferred that the amount of C_4_AcH_11_ formed during hydration reaction is not directly correlated with the amount of added CaCO_3_.

Additionally, since the XRD results showed that the 10 Cc specimen exhibited the highest crystallinity of C_4_AcH_11_, it suggests that the inhibitory effect on CAC conversion increases with the crystallinity of C_4_AcH_11_. This inference is supported by the comparative analysis of the XRD results.

The current study is limited by lab scale experiments and the specific range of CaCO_3_ content tested. Scalability of the manufacturing process and the economic feasibility of using CaCO_3_ in large-scale production have not been fully explored. Additionally, long-term durability and performance of these materials under varying environmental conditions need further investigation. Furthermore, the ability of CAC-based cementitious materials containing CaCO_3_ to form and maintain complex geometries requires additional validation to ensure the applicability of this technology to intricate and detailed designs. Future research should focus on optimizing CaCO_3_ content to achieve the best possible balance between material properties and cost-effectiveness. It should also explore the scalability of the BJAM process for industrial applications and assess the economic viability of large-scale production. Additional validation of the technology for producing complex geometries will be crucial in confirming its suitability for advanced casting manufacturing applications. Furthermore, since potential applications such as catalysts and casting molds require high thermal resistance, future research should include comprehensive heat resistance testing to ensure the materials meet the necessary performance standards for these high-temperature environments.

## Figures and Tables

**Table 1:** Prepared CAC based cementitious powder mixture and calculated w/c ratio for BJAM.

Specimen	s-SiO_2_ (wt.%)	CaCO_3_ (wt.%)	CaAl_2_O_4_ (wt.%)	C_12_A_7_ (wt.%)	w/c Ratio
Control	80	-	16	4	0.477
10Cc	70	10			
15 Cc	65	15			
20 Cc	60	20			

**Table 2:** Powder bed binder jet 3D printer specification.

Properties	Spectrum Z510
Printhead/Operating type	HP 11/Thermal inkjet
Resolution	600 × 540 dpi
Build Size(W × D × H)	254 × 356 × 203 mm^3^
Recoating type	Roller-spreading
Printing speed	100 mm/s
Layer thickness	102 μm
Activator	97% Di water

**Table 3:** Precise particle size distribution results of s-SiO_2_, CaAl_2_O_4_, C_12_A_7_, CaCO_3_ for BJAM.

Materials	d_10_ (μm)	d_50_ (μm)	d_90_ (μm)
s-SiO_2_	6.33	31.2	107
CaAl_2_O_4_	0.98	10.7	179
C_12_A_7_	1.16	12.1	92.4
CaCO_3_	1.98	19.3	38.3

**Table 4:** Measured powder bed density and standard deviation calculation results of sections 1–9.

Section	Powder Bed Density (g/cm^3^)			
	Control	10 Cc	15 Cc	20 Cc
1	1.31	1.47	1.50	1.52
2	1.32	1.45	1.51	1.53
3	1.35	1.48	1.50	1.50
4	1.32	1.46	1.52	1.49
5	1.35	1.49	1.52	1.53
6	1.33	1.49	1.51	1.51
7	1.31	1.48	1.50	1.52
8	1.34	1.46	1.50	1.53
9	1.32	1.46	1.49	1.51
Avg	1.33	1.47	1.51	1.52
Standard deviation	0.016	0.015	0.010	0.014

## Data Availability

The data presented in this study are available on request from the corresponding author.
